# Measurement Methods of the Thermal Resistance of Materials Used in Clothing

**DOI:** 10.3390/ma16103842

**Published:** 2023-05-19

**Authors:** Dubravko Rogale, Snježana Firšt Rogale, Željko Knezić, Nikolina Jukl, Goran Majstorović

**Affiliations:** 1 Department of Clothing Technology, University of Zagreb, Faculty of Textile Technology, 10000 Zagreb, Croatia; dubravko.rogale@ttf.unizg.hr (D.R.); nikolina.jukl@ttf.unizg.hr (N.J.); 2 Department of Textile Design and Management, University of Zagreb, Faculty of Textile Technology, 10000 Zagreb, Croatia; zeljko.knezic@ttf.unizg.hr; 3 Department of Physics and Materials, Faculty of Technical Sciences Čačak, University of Kragujevac, 32102 Čačak, Serbia; goran.majstorovic@ftn.kg.ac.rs

**Keywords:** textiles, clothing, thermal resistance, hot plate, multi-purpose differential conductometer

## Abstract

This paper describes methods for evaluating the thermal properties of textile materials, clothing composites, and clothing using an integrated measurement system that includes a hot plate, a multi-purpose differential conductometer, a thermal manikin, a temperature gradient measurement device, and a device for measuring the physiological parameters of the human body during the exact evaluation of garment thermal comfort. In practice, measurements were taken on four types of materials widely used in the production of conventional and protective clothing. The measurements were carried out using a hot plate and a multi-purpose differential conductometer, determining the thermal resistance of the material both in its uncompressed form and when a force was applied that was ten times greater than that needed to determine its thickness. Using a hot plate and a multi-purpose differential conductometer, thermal resistances of textile materials were assessed at different levels of material compression. On hot plates, both conduction and convection had an impact on thermal resistance, but in the multi-purpose differential conductometer, only conduction did. Moreover, a reduction in thermal resistance was observed as a result of compressing textile materials.

## 1. Introduction

Advances in measurement methods and devices for measuring thermal resistance through textile materials, garment composites and clothing have occurred in clothing engineering [1,2]. Thermal resistance is determined by experimental measurements and theoretical discussions. Both techniques highlight the exceptionally high complexity of thermal transmittance of textile materials since textile materials are exceedingly inhomogeneous materials beginning with non-uniform diameters and densities of textile fibres, spinning of fibres into yarns that are likewise inhomogeneous. Yarns are used to make textile fabrics (woven and knitted fabrics) of various constructions and homogeneities.

In clothing factories producing conventional and protective clothing to protect against very high or low temperatures (for the army, police, mountain rescue, postal service, fire department, etc.), testing the thermal properties of the incorporated materials is still rare, despite this being critical to the realization of clothing with specific thermal characteristics [3,4,5,6,7].

Most authors agree that thermal properties are most affected by entrapped air in fibres and yarns or by contained air in woven and knitted fabrics. Air gaps or air layers have been observed in clothing composites, as well as larger gaps and air layers in clothing, which significantly affect thermal properties [8,9,10].

Thus, according to Wilson et al. [11], research and content are related to (I) air contained in fabrics, air layers between fabric layers and air layers between fabric layers and the body, (II) garment shapes and the presence and influence of different fasteners and openings, and (III) the effects of wind and body movements of the wearer. In addition to these factors, the impact of body posture and airflow within the garment’s microclimate, as well as air exchange between the garment’s microclimate and the ambient air, will be investigated. The indicators for changes in thermal resistance are limited to the change in airflow in the microclimate of the garment or the change in energy required to keep the hot plate or thermal manikin at a constant temperature.

Woven and knitted fabrics that are essentially two-dimensional are transformed into 3D clothing by constructing and then assembling garments. In addition to measuring size and appearance, the thermal insulation properties of finished garments are also evaluated in terms of thermal resistance. The size and shape of the air layers, as well as their impact on thermal resistance, vary depending on factors such as the physical properties of the textile fabric, the clothing configuration such as body posture and the degree of coverage of the human body with clothing, wearer activity, and environmental conditions.

This review is divided into three major sections: (I) papers dealing with air in textile fabrics, between layers of integrated textile materials, between layers of integrated textile materials and the human body, and with the airflow in the microclimate of the garment relative to the ambient air [8,9,10]; (II) published research on clothing design issues, including body posture, clothing fit, and the availability and effectiveness of clothing fasteners and openings [12,13,14,15,16]; (III) influence of air flow (wind) in the environment on body movements [17,18].

All types of textile yarns and textile fabrics contain textile fibres and air entrapped within them, with the fibres being dominant in terms of mass and visibility and the air being dominant in terms of volume. For instance, the percentage of air in woven textile materials ranges from 60% to 90%, that in knitted fabrics ranges from 85% to 95%, and that in products such as quilted blankets ranges from 95% to 99% [19]. 

Since the thermal conductivity of fibres is 5–20 times higher than that of air [20], air entrapped inside yarns, textile fabrics, and garments contributes significantly to total thermal resistance.

Given that the discovered impact of entrapped air in textile materials contributes considerably to thermal resistance, it can be deduced that material thickness can be utilized for assessing thermal resistance. However, the thickness of the material is only acceptable for assessment if it is reliably measured because thickness measurements need compressibility in the measuring sample. Bogaty [21] examined the compressibility of the measuring sample during the measurement of a material’s thickness and discovered a certain nonlinearity. The linearity between thickness and thermal resistance ratio was also investigated for multiple layers of lightweight breathable material with air layers in between, and a non-linear dependence was found [22].

It is possible for air layers to form below, between, and above the surface of textile fabrics, which can impact on the thermal resistance of multilayer materials. The properties of air layers are determined by their thickness, shape and distribution. The geometry of the surface changes when a 2D textile product is wrapped around a human body, and as a result, the formation of layers next to one another may result in more air becoming trapped between the layers of the material which, generally speaking, is not usually distributed evenly. The textile covering is usually inhomogeneous and the distance between the textile layers can vary between different points of the covered body. 

The thermal properties of textile materials with contained air or with formed air layers have been investigated using various laboratory techniques [23].

As the size of air layers increases, the relationship between thermal resistance and number of layers becomes nonlinear [22,24].

Wilson et al. [11] demonstrated that air layers and the placement of layers inside the garment composite affect the value of thermal resistance. Air layers increase thermal resistance by 5–50%, and even small air layers can have an effect on resistance [24,25]. Greater thermal resistance can be better achieved by using multiple small air spaces between the layers of the garment composite than by using a larger air space of consistent thickness.

A number of authors have attempted to describe variables influencing the distribution and thickness of airspaces inside garment composites, as well as their thermal resistance. Variables include (I) material parameters (density, mass per unit area, fibre fineness, number of yarns and twisting, crimping, and compressibility) [26,27]; (II) manufacturing procedures (design of surface features and applications of finishing technologies) [28]; (III) garment design (including fit and composites), garment surface and geometric effects [29]; (IV) conditions of use (such as environmental conditions, airflow, and the intensity and type of physical activity of the clothing wearers) [30,31]. 

Most published test methods aimed at measuring thermal resistance through textiles are designed to minimize or specify the air space. Some authors increased the number of air spaces in order to get more realistic conditions.

On the garment and in garment composites, air gaps may be irregularly shaped, of varying thicknesses, distributed in various ways, oriented either horizontally, at an oblique angle or vertically, and may be either closed or open to the surrounding environment.

Whether the airspace is closed or open to the environment, the convection currents in the airspace are influenced by the orientation and thickness of the air layers. The differences in heat losses across the layers also depend on the orientation of the layers, such as heat transfer through horizontal airbags.

The complexity of the terms dealing with the thermal properties of textiles, clothing composites and clothing is also reported by Xu et al. They demonstrate very clearly the problems with measuring on the hot plate when the material sample is put directly on the test plate and, in principle, air gaps can be eliminated. Airbags occur between the thermal manikin and the layers of the garment composite when clothing composites are tested on a thermal manikin. As a result, significant variations in measurements emerge. Air has excellent insulating properties with a thermal conductivity of 0.026 Wm^−1^K^−1^ at ambient temperature. The thermal conductivity of airbags can fluctuate inside air gaps owing to airflow between the body and clothing, clothing and the external environment, the chimney effect [32], the pumping effect [33] (air displacement resulting from sporadic mechanical movements of the body limbs), natural convection, close contact with the skin and fabric surface, irregularly shaped air gaps that are internally caused by the weight of clothing or externally caused by pressure force, clothing construction, the wearer’s body posture, ease allowances, and other factors.

Measurements show that an air gap of about 5 mm improves insulation performance by 0.2 to 0.5 Clo [34,35].

These measurement results also confirm theoretical discussions on the contribution of air gaps to the overall thermal resistance of garment composites. Notably, 3D body scanning revealed that when a garment is placed on a thermal manikin, the layer of air that forms around the body and the surface of the garment can be as thick as 85 to 100 mm. Moreover, air gaps exist not only between the skin and the inside of the garment, but also between the layers of the garment composites. Therefore, thermal resistance measurements of garment composites on a hot plate may not accurately reflect the performance of garment composites in clothing. Furthermore, the airflows surrounding the measuring sample on the hot plate and on the manikin are determined by various speeds in accordance with the standard (1 ms^−1^ in a hot plate and 0.4 ms^−1^ in a thermal manikin). Therefore, the flow speeds must be equalized in order for the results to be comparable based on this parameter.

Regardless of the highly exact and reliable results acquired by various measuring methods of assessing thermal qualities, the subjective sense of wearing garments are still required [36].

## 2. A Newly Developed Integrated Method of Measuring the Thermal Properties of Clothing

This paper describes a newly developed integrated measuring system for thorough evaluations of the thermal properties of clothing. The system consists of five measuring methods and devices (hot plate, multipurpose differential conductometer, thermal manikin, device for measuring temperature gradients, device for measuring physiological parameters of the human body in the precise evaluation of the thermal comfort of clothing) that have been developed and calibrated, patented and/or used by the authors of this paper. To measure the thermal properties of textile materials used in clothing composites and garments, a measuring system and associated procedures are installed at the University of Zagreb Faculty of Textile Technology in the Laboratory for Thermal Insulation Properties of the Department of Clothing Technology.

**Figure 1:**
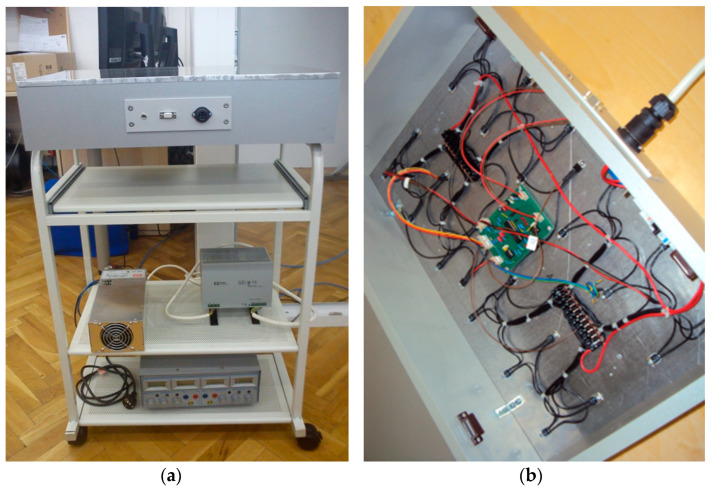
Constituent elements of hot plate: (**a**) external appearance; (**b**) interior with point heaters, temperature sensors and microprocessor system.

The guarded hot plate, Figure 1, consists of the measuring system and the temperature regulation system for the measuring area of the measuring device which is based on the electronic power control, the so-called PMW (pulse width modulation) technology. The measuring unit is a rectangular aluminium plate, 10 mm thick, connected to a metal block containing electric heating elements. The measuring area (0.4 × 0.6 m) is surrounded by an insulating thermal shield designed to prevent lateral heat loss from the sides of the gauge and fabric edges. The heating elements, located below the measuring surface, conduct heat to the measuring surface while preventing heat loss from the bottom of the measuring unit. This device design conducts heat upward only in the direction of the specimen thickness [37].

**Figure 2:**
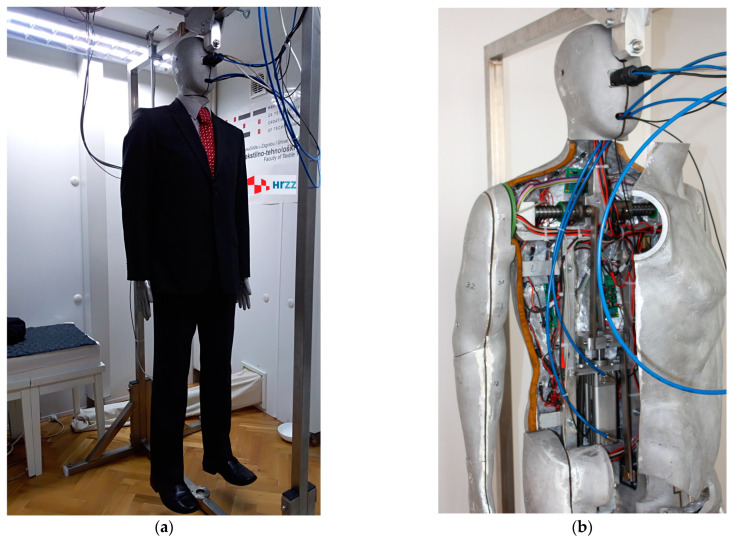
Thermal mannequin: (**a**) in climactic chamber with hot plate; (**b**) interior of thermal mannequin.

A segmented metal mould anatomically designed to simulate the human body, called the thermal mannequin, consists of 24 human body segments with built-in electric heaters, temperature sensors, 14 microcontroller interfaces, and a pneumatic system for arm and leg movements, as shown in Figure 2. The thermal mannequin is used to determine static and dynamic measurements consistent with the simulation of human walking.

Once the clothing is placed on the thermal mannequin, the determination of its thermal properties under dynamic conditions is performed in such a way that simulates the wearer’s gait, with both arms and legs moving in opposite phase. The limbs are moved using a pneumatic linkage system built into the thermal mannequin. The movement speed of the limbs can be varied over a wide range and precisely adjusted by the air damper to achieve a movement speed of 45 ± 2 double steps/min and 45 ± 2 double arm movements/min for walking, which is in accordance with the standard EN ISO 15831:2004 [38]. The method of control, regulation, measurement and calculation of thermal systems on the garment was introduced using a segmented metal casting modelled after the human body, with the possibility of activating and deactivating all segments (of the entire casting) or any group of segments of the casting, and of introducing and setting measurement parameters in accordance with the standards for experimental research.

When stable environmental conditions (temperature, relative humidity and air velocity) are achieved in the climatic chamber, the value of the device constant of the guarded hot plate and/or thermal mannequin should be determined, and can be obtained according to the following equation:

(1) $$R_{ct0}=\frac{\left(T_{s}-T_{a}\right)\cdot A}{H_{0}}$$

where: *R_ct_*_0_—resultant total thermal resistance of the measuring device including the thermal insulation of the boundary air layer, m^2^KW^−1^; *A*—total surface area of the hot plate/thermal manikin, m^2^; *T_s_*—mean skin surface temperature of the hot plate/thermal manikin, °C; *T_a_*—air temperature within the climate-controlled chamber, °C; and *H*_0_—total heating power supplied to the hot plate/ thermal manikin, W.

The clothing composite under test is placed on a hot plate after determining the resulting total thermal resistance of the gauge and measuring the heat flow through the test specimen after new stable conditions have been reached. The evaluation of the thermal properties of the clothing using the thermal mannequin is performed by placing the selected clothing or ensemble around its body in either static or dynamic mode of operation. In dynamic measurement, the thermal mannequin simulates the wearer walking, with both the legs and arms of the manikin moving in phase reversal, with a specified number of movements per minute and a specified stride length. The measurements can be performed under static or dynamic environmental conditions simulated in the climatic chamber. After determining the thermal comfort, which can be seen from the stabilization of the parameter values (numerical and shown in diagrams), measurements are made and the thermal resistance is calculated using Equation (2) [39]:

(2) $$R_{ct}=\frac{\left(T_{s}-T_{a}\right)\cdot A}{H}-R_{ct0}$$

where: *H*—location where the electrical power required to maintain the temperature of the measuring surface on which the measurement sample is positioned is provided.

A multi-purpose differential conductometer [40] is used to measure: (I) The resistance to the passage of heat or thermal conductivity at one or more cutting positions on the garment to be produced. In this way, the success of choosing is tested for the built-in materials, and the type and number of layers in the garment or composite that will be built into in the garment. This invention also enables measurements on a pre-fabricated garment when it is necessary to determine thermal properties at specific locations in a non-destructive manner. (II) The thickness of garments or composites at different locations, whereby thickness measurements can be made when the measuring sample is loaded with a specific pressure (as in standard measurements of the textile materials thickness with the so-called preload) or not. (III) Compressibility of measured clothing parts, i.e., compressibility of clothing or laminate parts under certain forces or pressures, and drawing functional diagrams of changes in clothing or laminate thicknesses with changes in compressive forces or specific pressures. (IV) Resistance to the passage of heat at different values of material compression or its thickness, which is often the case when wearing clothing or objects on clothing (e.g., backpacks). (V) Differential temperatures between the individual layers in the garment in free and compressed state whereby the garment constructor obtains information on the success of applying the thermal properties of each of the designed layers in the garment and on the possible need for correction.

According to expert knowledge and a search of the available literature, there is no similar device or measurement system to the device designed, calibrated, installed and patented at the Faculty of Textile Technology, which as a multi-purpose differential conductometer for textile composites and clothing.

**Figure 3:**
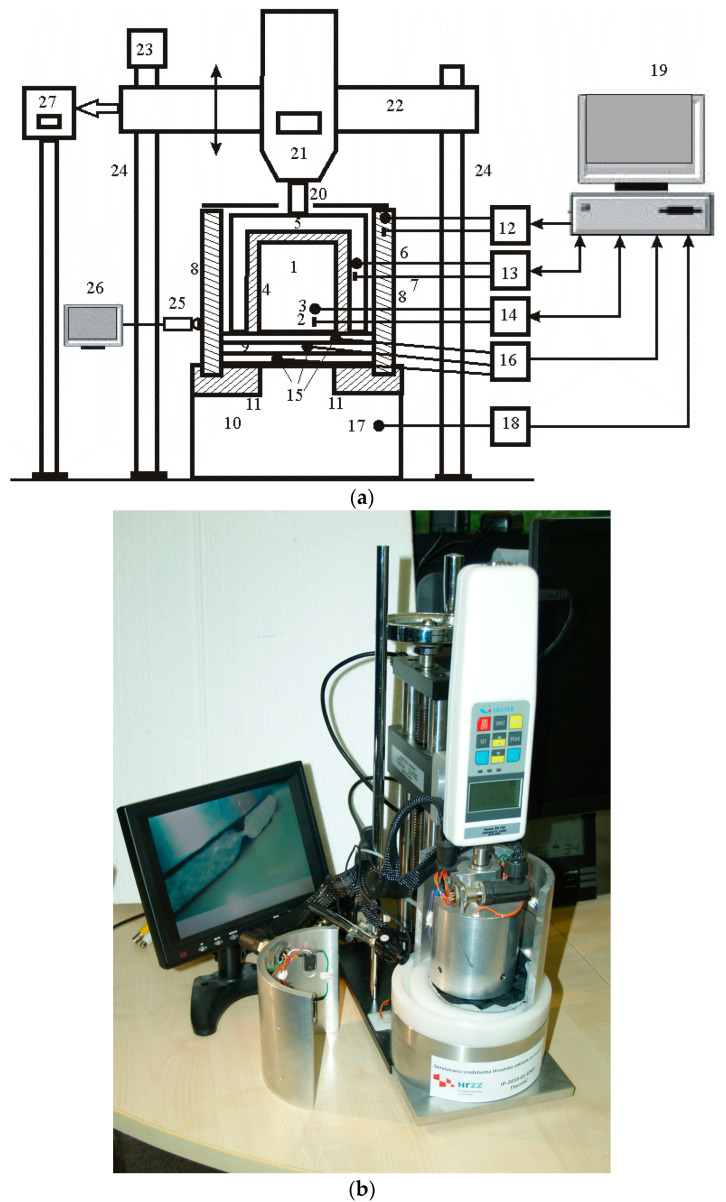
Multi-purpose differential conductometer: (**a**) schematic diagrams; (**b**) realized device.

A multi-purpose differential conductometer, Figure 3, consists of a measuring cylinder (1) with heaters (2) and temperature sensor (3). The measuring cylinder is thermally insulated with Teflon (4) and is located in the first heat holder (5), which is heated by heaters (6) and has a temperature sensor (7). The second heat holder (8) is placed on the measuring base (10) and the Teflon ring (11). The heat holders have measurement control systems (12 and 13), and the measuring cylinder has a measurement control system (14). These systems are used to measure steady-state maintenance performance. Sensors (15) between layers measure differential temperature with assembly (16), measurement base temperature with assembly (17), and measurement base temperature with assembly (18). The data is entered into the computer (19), which contains software for calculating the thermal properties of samples in the relaxed or compressed state. The compressed state is achieved by means of a support (20), a dynamometer (21), a console (22) and a movable mechanism (23) with guides (24) for connecting the measuring cylinder with a sample determined by the video camera (25) and a monitor (27) or a dynamometer press with displacement measurement data (27).

If there is no contact or no reaction of the textile fibres on the upper contact plate, that is, no contact heat transfer, the conductometer displays the value zero. At zero load, there is no contact between the material and measuring cylinder, so thermal flux cannot be established.

When stable conditions are established—i.e., the heat flow from the measuring cylinder to the measuring base is in steady state—the temperature of the measuring cylinder, the measuring base, and the electric power supplied to the measuring cylinder required to maintain the steady state are read, and the thermal resistance is determined using the Equation (1), where *H*_0_ is total heating power supplied to the measuring cylinder, W.

On establishing thermal equilibrium (steady state), the power required to maintain thermal equilibrium of the measuring cylinder is calculated using the following equation:

(3) $$H_{0}=\frac{U_{g}^{2}\cdot P_{PWM}}{R_{g}}$$

where: *U_g_*—the voltage of the stabilized source that supplies the non-inductive point heaters of the multi-purpose differential conductometer; *P_PWM_*—the ratio of the *PWM* at the interface output; and *R_g_*—the total electrical resistance of the non-inductive point heaters.

The general expression for calculating the thermal resistance in one or more layers of the composite clothing *R_ct_* using the parameters shown, after restoring the stationary state (*T_s_* = const., *T_a_* = const., *H*_0_ = const. and q = const.), takes the form of equation:

(4) $$R_{ct}=\frac{\left(T_{MC}-T_{MB}\right)\cdot A_{MC}\cdot R_{g}}{U_{g}^{2}\cdot P_{PWM}}$$

where: *A_MC_*—total surface area of the measuring cylinder, m^2^; *T_MC_*—mean skin surface temperature of the measuring cylinder, °C; *T_MB_*—mean skin surface temperature of the measuring base.

**Figure 4:**
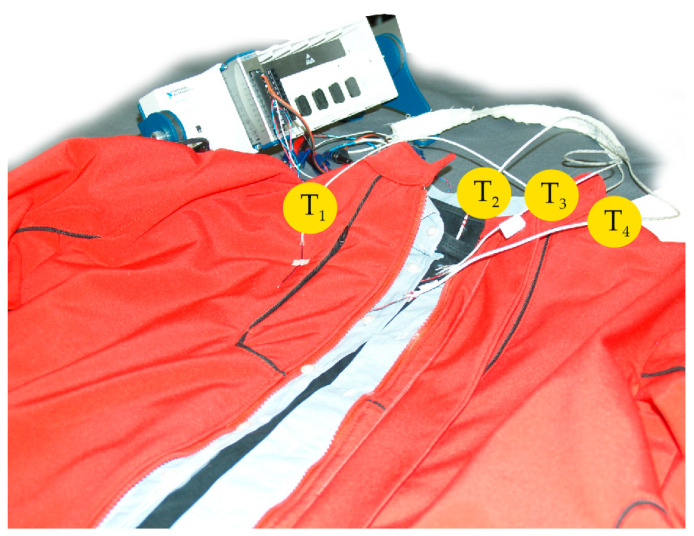
The measurement of the temperature gradients in clothing.

The measurement of the temperature gradients [41,42] also only begins once steady state has been established, and proceeds in such a way that the temperature values of the four sensors (T_1_ to T_4_, Figure 4) placed between the four clothing layers are outputted to the measuring amplifier and temperature compensator at the cold end of the thermocouples, and then to the measuring computer, as shown in Figure 4. 

**Figure 5:**
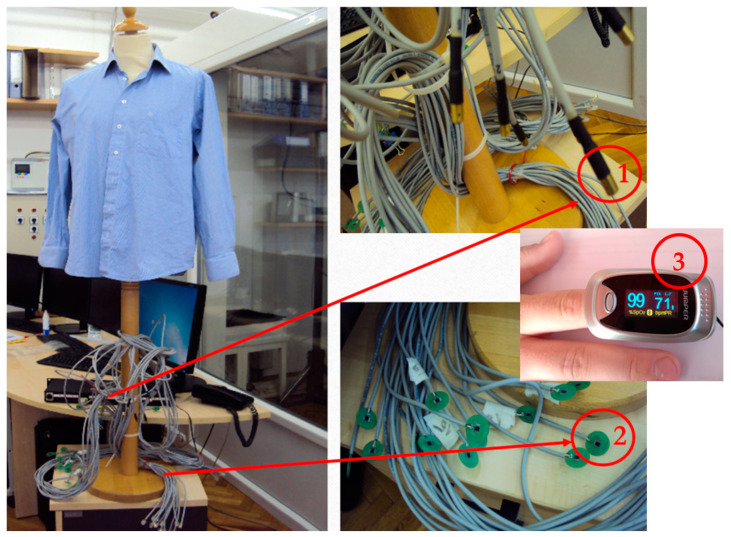
Measurement system and method for assessing the physiological properties of the human body by accurately evaluating the thermal comfort of clothing using temperature (**1**) and humidity (**2**) sensors and pulse oximeters (**3**).

Steady state for the measured samples can be achieved in about 7 min. Due to the variation in measurement conditions, measurements of all measurement samples should start after 10 min. After that, measurement of the temperature gradients can take place every minute for 20 min, after which average values of the temperatures are calculated, similar to the thermal mannequins, according to EN ISO 15831:2004 [38]. If using a measuring device without a cover, it is desirable for this to be placed in the air-handling unit, together with the thermal mannequin, in order to ensure the same measurement conditions; alternatively, the measuring device can be constructed as a separate device with components determining the condition of the miniature air-handling unit under the cover. Figure 5 shows a new measuring instrument developed by the Faculty of Textile Technology to assess the physiological properties of the human body by accurately assessing the thermal comfort of clothing.

The measurement method for measuring the physiological parameters of the human body in the exact assessment of thermal comfort of clothing was determined according to the EN ISO 9886:2004 standard [43]. Thermal comfort when wearing garments can be measured by the wearer’s subjective expression or by precise measuring of physiological parameters: skin temperature (as measured using the method of four, eight, or fourteen points from which the mean weighted skin temperature is obtained), relative skin moisture (sweating), and heart rate. 

The mean weighted skin temperatures are calculated according to the following equations [43,44]:

(5) $$T_{sk4}=0.28\times T_{2}+0.28\times T_{5}+0.16\times T_{7}+0.28\times T_{12}$$

(6) $$T_{sk8}=0.07\times T_{1}+0.07\times T_{3}+0.175\times T_{4}+0.175\times T_{5}+0.07\times T_{6}+0.05\times T_{7}+0.19\times T_{10}+0.20\times T_{13}$$

(7) $$T_{sk14}=0.07\times T_{1}+0.07\times T_{2}+0.07\times T_{3}+0.07\times T_{4}+0.07\times T_{5}+0.07\times T_{6}+0.07\times T_{7}+0.07\times T_{8}+0.07\times T_{9}\;+0.07\times T_{10}+0.07\times T_{11}+0.07\times T_{12}+0.07\times T_{13}+0.07\times T_{14}$$

where: *T_sk_*—mean weighted skin temperature, °C; and *T*—temperatures, °C on: forehead (*T*_1_), neck (*T*_2_), right shoulder (*T*_3_), left upper chest (*T*_4_), right scapula-back (*T*_5_), left arm (*T*_6_), left hand (*T*_7_), right abdomen (*T*_8_), left paravertebral (*T*_9_), right anterior thigh (*T*_10_), left posterior thigh (*T*_11_), right shin (*T*_12_), left calf (*T*_13_), right instep (*T*_14_).

The human physiological parameter measuring device for accurately assessing the thermal comfort of clothing consists of four modules: (I) skin temperature measurement module, (II) heart rate measurement module, (III) module for measuring the relative humidity of the skin (sweat) and (IV) module for measuring the temperature between layers of clothing.

This device reduces the subjectivity of the assessment and highlights the importance of accurate measurements.

The module for measuring the skin temperature of the wearer of the clothing system can measure the skin temperature in four, eight, or fourteen points and calculates the weighted average skin temperature according to the EN ISO 9886:2004standard [43]. In order to accurately assess the thermal comfort of clothing in a hot environment by measuring the physiological characteristics of the human body, the temperature is measured at four points on the body in accordance with Equation (5); for neutral climatic conditions—at 8 points according to Equation (6); and for a cold environment, temperature measurements are taken at 14 points on the body according to Equation (7). Each measuring point has a specific coefficient.

The module for measuring the heart rate of the wearer of the garment system is also defined by the ISO 9886:2004 standard [43].

The module for measuring the relative humidity of the skin (sweating) of the wearer of the clothing system and the presentation of the measurement results, as well as the module for measuring the temperatures between the individual clothing layers to determine the percentage of the heat insulation effect of each layer in the clothing system, are an addition to the standard method, and thus the modified method allows for more scientific understanding of heat transfer between the wearer’s body (through clothing composites integrated into the clothing) and the environment.

The methods and equipment described can be used to determine the thermal properties of all building materials in clothing (textile materials, knitted fabrics, nonwovens, etc.), their composites, and all types of clothing for various conditions and types of heat transfer.

## 3. Materials and Methods

The choice of materials for the production of test samples is based on many years of practical experience, working as a chief technologist and technical director of various production systems for the production of professional, protective and other special clothing. As a result, essential professional expertise was obtained, as well as access to specific cutting-edge integrated materials for protective clothing that are not available through traditional commercial channels. 

The necessary technical properties of the selected sample production materials were analysed, followed by the thermal resistance of the material itself in flat shape using the hot plate method.

First, the technical qualities of the raw materials utilized to make the sample were examined. 

**Figure 6:**
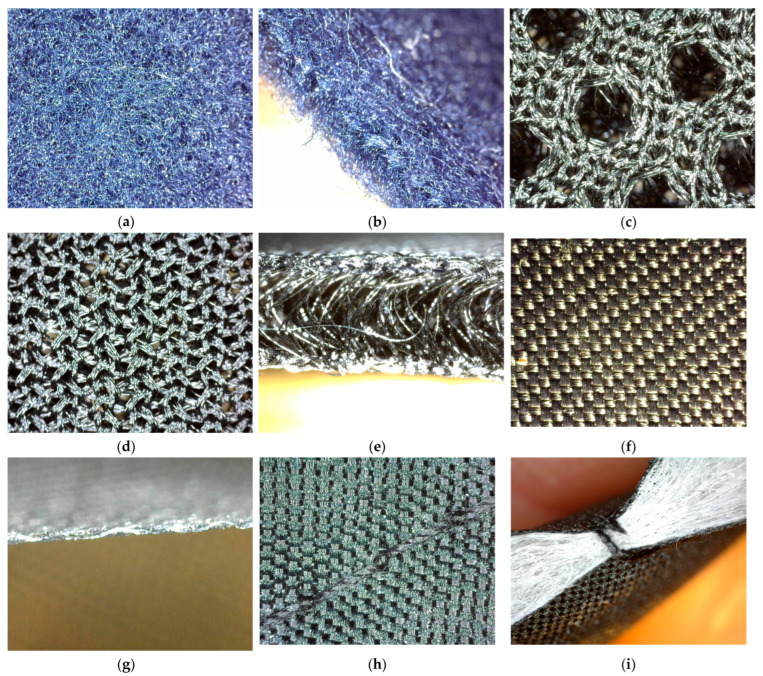
Microscopic images of integration materials: (**a**) front and back side of fleece material (M1); (**b**) cross-section of fleece material (M1); (**c**) front side of spacer material (M2); (**d**) back side spacer material (M2); (**e**) cross-section of spacer material (M2); (**f**) front and back side of lining material (M3); (**g**) cross-section of lining material (M3); (**h**) front and back side of double-faced, diamond-shaped quilted lining (M4); (**i**) cross-section of double-faced, diamond-shaped quilted lining (M4).

The selected integration materials include fleece-material (M1), spacer material (M2), lining material (M3) and double-faced, diamond-shaped quilted lining (M4); their technical characteristics are presented in Table 1. Figure 6 shows microscopic images of these materials.

**Figure 7:**
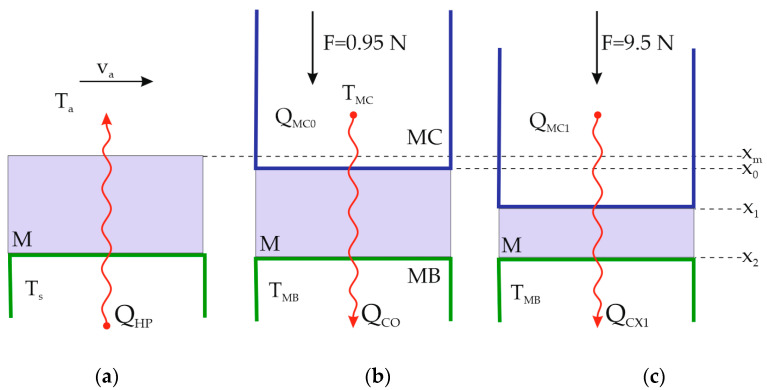
Measurement of the thermal resistance of the material: (**a**). on hot plate; (**b**) on multi-purpose differential conductometer in the initial state (F = 0.95 N) (**b**,**c**). in the compressed state (F = 9.5 N).

Figure 7 shows the typical measurement of the thermal resistance of the material on a hot plate (Figure 7a) and the measurement of the thermal resistance of the material with a multi-purpose differential conductometer in the initial state (F = 0.95 N) (Figure 7b) and in the compressed state (F = 9.5 N) (Figure 7c).

A heat flux *Q_HP_* is generated when the hot plate reaches temperature *T_s_*. This flux travels through the material M and is then released at temperature *T_a_* and flow rate *v_a_* into the surrounding air. The material sample, shown in Figure 7a, is *x_m_* thick and is not compressed.

When measuring thermal resistance with a multi-purpose differential conductometer, as shown in Figure 7b, a heat flux Q is created from the *T_MC_* temperature measuring roller through the material M to the MB measuring base with *T_MB_* temperature.

To determine the material thickness, the measuring cylinder presses it with a force of F = 0.95 N in accordance with EN ISO 5084:1996 [45], resulting in a small amount of flattened material. The height of the roller under this force is the value *x*_0_, which is the initial reference position for generating and measuring the heat flux *Q_C_*_0_.

Even greater material compression is attained when the compressive force of the measuring cylinder is increased by a factor of 10, to 9.5 N, with the cylinder positioned at position *x*_1_ (compression displacement), where the heat flow *Q_MC_*_1_ is created and measured. The measuring sample is removed at the end of the measurement, and the measuring cylinder MC is lowered to the height *x*_2_ as shown in Figure 7c. Thus, Equation (8) can be used to calculate the compressibility:

(8) $$S=\frac{x_{1}}{x_{2}}\times 100,\%$$

Integration materials for thermal inserts and lining materials must meet the professional criteria of vapour permeability, water resistance and air tightness. The measured parameters and their results are directly related to the knowledge of the properties of the integration materials intended for the protective clothing and affect the future condition of the systems inside the garment. 

Measurement of the thermal resistance of materials used in clothing was carried out using a hot plate, shown in Figure 2. The hot plate was developed at the University of Zagreb Faculty of Textile Technology.

## 4. Results and Discussion 

The results of the measurement of thermal resistances of the materials used in the clothing carried out with the hot plate according to Figure 7 are shown in Table 2. In Table 3, the measurements of the thermal resistance with the multi-purpose differential conductometer are shown.

The selected integration materials of fleece material (M1), spacer material (M2) and double-faced, diamond-shaped quilted lining (M4) are outstanding thermal insulators. These materials have excellent water vapour permeability, as can be seen in Table 1. The thickening of the fibres within the structure of the double-faced, diamond-shaped quilted lining system results in a decrease in water vapour permeability (2577.9 gm^−2^day^−1^). The results obtained indicate adequate wearing comfort, as sweat transport is made possible by the integration of these materials.

Air permeability tests show different results for some types of materials. Due to their structure, spacer materials have very high air permeability (average 91.66 m^3^m^−2^min^−1^ and up to immeasurably high values), whereas double-faced, diamond-shaped quilted materials exhibit restricted air permeability (8.8 m^3^m^−2^min^−1^). The cause for the decreased air permeability is the development of a sandwich consisting of single or double lining fabric as the padding, inside which are the polyester fibres, within the diamond-shaped quilt placed on a specific surface, giving the effect of a filled insert with voluminous fibres. The double-faced, diamond-shaped quilted lining construction limits air exchange and retains air inside the insert or chamber, so creating an air barrier.

From a technical point of view, there are two ways of considering the air. The first is that air is free to circulate inside the chambers of spacer material and exchange with the environment, whereas the second is that air entrapped inside the chambers of double-faced, diamond-shaped quilted material has reduced internal mobility and exchange with the environment.

In terms of heat-insulating material qualities, the exception is fleece material, which has very good vapour permeability (4341.8 gm^−2^day^−1^). Due to the construction of the fleece material, the water vapour is not bound, but guided directly through the material. The fleece material has a maximum moisture absorption of up to 4%, and because the fibres are intertwined and amorphous, there is less air exchange; therefore, these qualities accentuate its thermal insulation capabilities.

Lining materials of polyester raw material composition (M3) are utilized to make the lower layers of the outer shell and the lower layers of the thermal insert. Those on the inside round out the garment system and provide comfort when worn. In principle, this material is not a thermal insulator.

Except for the lining, all of the integration materials have a very noticeable material thickness characteristic. Thermal resistance is exhibited by fleece materials, spacer materials, and double-faced, diamond-shaped quilted linings, which prevent heat passage. They are distinguished by a decrease in electric power as well as a decrease in the thermodynamic balance between the power required to maintain the constant temperature on the surface of the hot plate and the heat radiated to the environment by the material being measured. As a result, the calculation has shown a favourable resistance to heat transfer, demonstrating the ability of the measured materials to provide thermal insulation.

A broad range of thermal resistance values can be seen in the established results of the tested thermal resistance on the hot plate (Table 2). The values of spacer (0.0153 m^2^KW^−1^) and fleece (0.0156 m^2^KW^−1^) materials are low. Spacer materials have extremely high air exchange values, even immeasurably so, and this contributes to heat exchange with the environment and a reduced thermal resistance value. Fleece materials with lower air permeability values have higher thermal resistance values than spacer materials, although a double-faced, diamond-shaped quilted lining with a polyester insert achieves adequate results.

Characteristic of the lining fabric is the fact that no decrease in electrical power was detected at the established thermodynamic power balance required to maintain a constant temperature of the hot plate surface and the heat radiated into the environment through the measured material. On the other hand, there was a rise in the amount of electrical power needed to keep thermodynamic equilibrium, and the calculation revealed negative thermal resistance (−0.0019 m^2^KW^−1^), indicating that the measured material loses its insulating capabilities and acquires heat conductivity properties.

The apparent anomaly observed may be accounted for by the fact that the thickness of the lining material is only a few tenths or hundredths of a millimetre, thereby lacking the characteristics of a thermal insulator, but instead exhibiting those of a comparatively efficient thermal conductor. Thus, the reason for this measurement result is that the test material lacks the required thickness or voluminosity, and as a result, lacks the capacity to retain air in its structure, which is a prerequisite for the determination of the thermal insulation properties of materials used in clothing.

During measurements, the fleece material (M1) achieved the maximum thermal resistance of 0.0156 m^2^KW^−1^ on the hot plate, while the spacer material (M2) achieved a slightly lower thermal resistance of 0.0153 m^2^KW^−1^. Due to the inadequate measurement of materials with protruding segments on the hot plate, the double-faced, diamond-quilted lining acquired a relatively low thermal resistance of 0.0871 m^2^KW^−1^. The double-faced, diamond-shaped lining has a bulge in the centre of the rhombus and channels running along the quilted seams. When measured on the hot plate, heat is thought to move down the quilted channels by convection, affecting the measured amount of thermal resistance. Higher values of thermal resistance for the same materials were obtained in the measurements with the multi-purpose differential conductometer. The conductometer simply measures the thermal contact conductivity between the outside and inside of the textile material, excluding heat transfer by means of convection. As a result, the thermal resistance values are higher than those of the hot plate. The impact of heat conduction through textile materials and convection in the gaps between textile fibres, yarns, warp and weft threads in woven fabrics or loops in knitted fabrics is significantly more noticeable with hot plates. The lining fabric (M3) had the lowest thermal resistance of 0.081 m^2^KW^−1^, which also decreases with ten times higher force when evaluating compressibility at 0.0076 m^2^KW^−1^. When measured, the double-faced, diamond-shaped lining has the highest thermal resistance due to its maximum thickness of 5.36 mm and the greatest volume with the compressibility of 58%. The design of the multipurpose differential conductometer prevents heat dissipation by convection flow along the quilted channels, which significantly affects the accuracy of the measurements and the accuracy of the measurement results. Because of its very great thickness, the spacer material (M2) also has a comparatively high thermal resistance. The fleece material (M1) has lower thermal resistance than the spacer material due to the high density of the textile materials, which results in relatively good thermal conduction, while spacer materials (M7) are dominated by integrated air chambers, which effectively improve thermal resistance. The lowest degree of compressibility, found in the spacer materials, was 21% at tenfold compression, while the double-faced, diamond-shaped quilted lining had a compressibility of 58%. Material compressibility is directly related to the decrease in thermal resistance, which is due to the increased compaction of textile fibres, yarns and fabrics, as well as reduced thickness, all of which increase thermal conduction through textile materials. 

According to measurements made using a multipurpose differential conductometer, the thermal resistance of a material can be reduced by 94–49% after being compressed 10 times compared to its original value while uncompressed. 

It is reasonable to anticipate that the thermal resistance measurements obtained from the hot plate and multi-purpose differential conductometer appear to be comparable and may yield comparable outcomes. Nonetheless, they are fundamentally different due to a significant reduction in thermal conductivity (resistance) detected in the conductometer, which assesses thermal contact conductivity. When measuring thermal contact conductivity, a pressure similar to that used when measuring the thickness of a textile sample is applied to the sample to reduce the thickness of the sample. Air layers between layers of textile materials, measurement surfaces of a multifunctional differential conductometer, and inside layers of composite garments are also removed. As the sample is compressed, the thermal resistance reduces further. This is because the entrapped air volume in the textile material reduces and the conductivity of the textile fibres, yarns, and constructions of woven or knitted fabrics becomes more significant.

## 5. Conclusions

This manuscript aims to present measurement methods and an integrated measurement system consisting of five measurement methods and devices developed and patented at the University of Zagreb Faculty of Textile Technology. For this reason, only preliminary results are presented, indicating large differences in the values of thermal properties that depend on the different types of heat transfer through textiles and clothing. Further research will be directed towards determining the thermal properties of textile materials, their composites and clothing in order to clarify the factors influencing heat transfer.

The high complexity of measuring thermal resistance through textile materials is evident when considering the obtained results and the application of the new measuring devices described in this paper. Because measurements on a hot plate could have an impact on two different types of heat transfer (conduction and convection), it is clear that not all influencing factors can be captured with this single piece of measuring equipment; by contrast, the conductivity meter measurement result is mainly determined by heat conduction. Similarly, the conductometer allows measurements of heat transfer at varied degrees of material compression, thickness, and particular pressures that affect the material. The type of materials from which textile fabrics are made, their construction and the entrapped air inside them have an important influence on thermal resistance. All of this implies that the measurement of thermal resistance must take into account the measurement methods used, an in-depth knowledge of the type and structure of the textile materials, as well as their raw material composition; in light of this, defining all the influencing parameters will be a scientific challenge for a long time to come. 

The conductometer is used to measure the thermal contact conductivity between two plates at different temperatures. It is therefore necessary to establish physical contact between plates and materials (woven fabrics, knitted fabrics, yarns and textile fibres within the fabric structure). To establish the contact, the material must be slightly compressed; for this purpose, it is necessary to choose the same specific pressure, which corresponds to the pressure applied to the textile material when measuring its thickness according to the standard and the method of measuring the thickness of textile materials.

The design of the multi-purpose differential conductometer avoids possible errors, especially the heat flow on the measuring cylinder. The measuring cylinder is fenced with a heat holder, which prevents heat flow to the side and upwards. Therefore, heat flow is possible only from the measuring cylinder to the measuring base. To prevent heat flow to the side for thicker materials and their composites, as well as for parts of clothing, another outer shield has been added.

The hot plate and the thermal mannequin are placed in the same climatic chamber and the measurements are performed under the same environmental conditions as specified by the standard EN ISO 15831:2004 [38]. The determination of thermal properties of textile materials and clothing composites on the hot plate is performed in turbulent flow, as it is on the thermal mannequin, unlike other hot plates where measurements are performed in laminar flow. This allows the determination of thermal properties of insulation materials in the development of clothing with predefined and desired thermal properties.

## Figures and Tables

**Table 1:** Overview of the analysed technical characteristics of the sample of the integration material.

Technical Characteristics	Fleece Material (M1)	Spacer Material (M2)	Lining Material (M3)	Double-Faced, Diamond-Shaped Quilted Lining (M4)
Raw material composition, %	Polyester 100	Polyester 100	Acetate 50 Viscose 50	Cover fabrics: Polyester 100 Lining: Polypropylene 100 Padding: Polyester 100
Mass per unit area, gm^−2^	298.8	315.9	69.6	Total: 278.3 Cover fabrics: 68.3 × 2 Lining: 12.4 Padding: 129.3
Vapour permeability, gm^−2^day^−1^	4341.8	3723.7	4575.75	2577.9
Air permeability, m^3^m^−2^min^−1^	19.33	91.66	13.99	8.8

**Table 2:** The results of the measurement of thermal resistance of the materials used in the clothing carried out with the hot plate.

Force [N]	Test Element	Fleece Material (M1)	Spacer Material (M2)	Lining Material (M3)	Double-Faced, Diamond-Shaped Quilted Lining (M4)
0	Thermal resistance, m^2^KW^−1^	0.0156	0.0153	−0.0019	0.0871

**Table 3:** The results of the measurement of thermal resistance of the materials used in the clothing carried out with multi-purpose differential conductometer.

Force [N]	Test Element	Fleece Material (M1)	Spacer Material (M2)	Lining Material (M3)	Double-Faced, Diamond-Shaped Quilted Lining (M4)
0.95	Thickness of the material, *x*_2_, mm	2.92	3.28	0.24	5.36
	Thermal resistance, *R_ct_*, m^2^KW^−1^	0.0172	0.1045	0.0081	0.2229
9.5	Compression displacement, *x*_1_, mm	0.67	0.69	0.09	3.11
	Compressibility, *S,* %	23	21	37.5	58
	Thermal resistance, *R_ct_* [m^2^KW^−1^]	0.0164	0.0853	0.0076	0.1095

## Data Availability

The data presented in this article are available upon request from the corresponding author.
